# Cost-effectiveness analysis of low density lipoprotein cholesterol-lowering therapy in hypertensive patients with type 2 diabetes in Korea: single-pill regimen (amlodipine/atorvastatin) versus double-pill regimen (amlodipine+atorvastatin)

**DOI:** 10.4178/epih/e2015010

**Published:** 2015-02-22

**Authors:** Ji-Hyun Park, Yong-Ho Lee, Su-Kyoung Ko, Bong-Soo Cha

**Affiliations:** 1 Pfizer Pharmaceuticals Korea Ltd., Seoul, Korea; 2 Department of Internal Medicine, Yonsei University College of Medicine, Seoul, Korea

**Keywords:** Atorvastatin calcium, Amlodipine besylate, Medication adherence, Low density lipoprotein cholesterol, Cost-effectiveness analysis

## Abstract

**OBJECTIVES:**

Single-pill combination therapy (amlodipine/atorvastatin) might be more effective than double-pill therapy (amlodipine+atorvastatin) in patients with diabetes and concomitant hypertension requiring statin therapy. We compared the cost-effectiveness of a single-pill with that of double-pill for control of low density lipoprotein cholesterol (LDL-C) levels, with the ultimate goal of cardiovascular disease prevention, in these patients using a cost-effectiveness analysis model that considered medication adherence.

**METHODS:**

Effectiveness was defined as the percentage (%) attainment of target LDL-C levels (<100 mg/dL) based on adherence for each therapy. Adherence was defined as compliance to medication (≥80% proportion of days covered). A systematic review of the literature was conducted to determine the proportion of patients who were adherent and target goal attainment based on adherence level. The annual medication costs were based on the adherence levels for each regimen. The average cost-effectiveness ratio (ACER) was calculated as the cost per % attainment of the target LDL-C level.

**RESULTS:**

The ACER for the single-pill regimen was lower than for the double-pill regimen (4,123 vs. 6,062 Korean won per 1% achievement of target goal). Compared with the double-pill, the medication costs were approximately 32% lower with the single-pill.

**CONCLUSION:**

A single-pill for reductions in LDL-C is cost-effective compared with double-pill in hypertensive patients with type 2 diabetes.

## INTRODUCTION

According to literature published in 2011, the prevalence of cardiovascular diseases (CVDs) is sharply increasing, as are the related mortality rates. This trend is reportedly related to the increasing rates of hypertension, diabetes, dyslipidemia, and obesity [1]. Furthermore, when these diseases are concomitant, the risk of CVDs increases. As such, proactive management of these risk factors is critical [2]. Particularly for patients with diabetes, the additional burden of hypertension and dyslipidemia significantly increases the risk of CVDs. Therefore, recent treatment guidelines recommend strict control of blood cholesterol levels (low density lipoprotein cholesterol [LDL-C] <100 mg/dL) in addition to effective blood pressure management to prevent CVDs [3,4].

In general, less than half of treated patients achieve and maintain the target blood pressure and cholesterol levels [5], which might be attributed to rates of medication adherence [6]. Medication adherence is a critically important factor influencing treatment outcomes and management of many acute and chronic health conditions [7]. Low adherence is reportedly ascribable to the prescription of multiple medications and complex medication regimens. Therefore, simpler regimens of single-pill might have improved medication adherence and subsequently treatment outcomes [8,9].

A blood pressure-lowering agent, amlodipine besylate (referred to as amlodipine), and a lipid-lowering agent, atorvastatin calcium (referred to as atorvastatin), which has a bio-profile equivalent to its parent compounds [10], combined in a single-pill significantly improves medication adherence rates [11]. Therefore, it is likely that single-pill will help patients reach their target blood pressure and cholesterol levels.

Because single-pill is generally less expensive than the double-pill, the overall economic burden of medications could decrease with the use of single-pill. However, the actual economic effects of potentially improved medication adherence need to be evaluated because patients with low medication adherence likely spend less on medications anyway and a low adherence rate would negatively influence the treatment outcomes. This study aimed to examine and compare the cost-effectiveness of a single-pill regimen (amlodipine/atorvastatin) with a double-pill regimen (amlodipine+atorvastatin) in patients with diabetes and concomitant hypertension who also require cholesterol management.

Cost-effectiveness analysis (CEA) is a form of economic analysis that helps identify rational methods to allocate resources for public health. It is designed to evaluate the relative costs and outcomes of specific medications while considering quality of life (QOL). The analysis compares the clinical usefulness with potential costs to identify an alternative that maximizes health for a given set of resource constraints.

## MATERIALS AND METHODS

The three main types of costs that are relevant in a CEA are direct costs (e.g., medical and non-medical costs) and indirect costs (e.g., productivity loss) [12]. Medical costs refer to expenses paid to utilize medical services (e.g., doctor and hospital visits, in-patient treatments) and purchase necessary medical equipment (e.g., medications). Non-medical costs include expenses that typically accompany medical costs, including nursing care fees, transportation fees, and other financial burdens placed on family members for patient care. Finally, productivity loss refers to the economic loss from treatment of illnesses or premature deaths resulting from illnesses. Direct medical costs are the most relevant in terms of an economic analysis. Cost evaluation methods used in economic analysis vary only slightly. However, depending on the definition of the outcome, cost-minimization analysis (CMA), CEA, cost-utility analysis (CUA), or cost-benefit analysis (CBA) can be used [13].

CMA is used to compare the costs when the clinical effectiveness of alternatives has proven to be equal. With this tool, the optimal option is the one with the lowest associated costs. CEA and CUA are used to evaluate the costs as well as the outcomes of alternatives when the outcomes are known to vary. In particular, CUA considers both the length of life and QOL by incorporating indices such as quality adjusted life years and healthy years equivalents. CBA is a quantitative tool that calculates intangible benefits of improved health and life extension in terms of dollar values [13].

This study utilized CEA to compare both the clinical effectiveness and costs of the treatment alternatives. For cost estimation, only the medication costs were considered based on the assumption that all other associated costs were equal. Additionally, the medication-related outcomes were assumed equal, and the patient’s medication adherence was considered the only factor potentially affecting this balance.

A decision analytic model is established for a comprehensive economic analysis that considers and integrates various data related to costs and effects. A decision tree model and Markov model are typically used for decision analytic models. This study employed the former, which calculates the expected costs and outcomes of each treatment alternative by integrating the data on potential health outcomes and costs.

A level of uncertainty is expected to accompany economic analysis because they integrate multiple research findings based on various assumptions. However, to verify and minimize the uncertainties, a sensitivity analysis is performed for the variables used to estimate costs and effects. In this study, sensitivity analysis were performed for medication adherence rates and target goal attainment rates based on adherence.

### Cost-effectiveness analysis model

**Figure 1.**
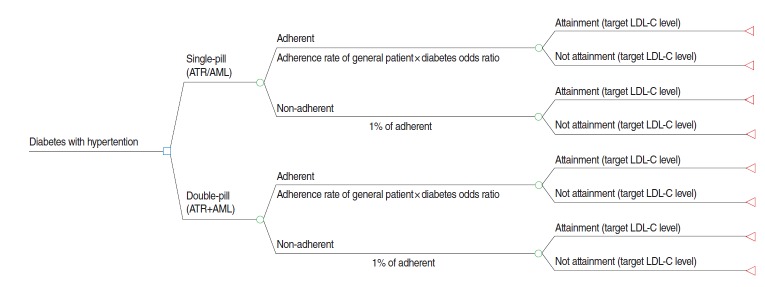
Cost-effectiveness analysis model. ATR, atorvastatin; AML, amlodipine; LDL-C, low density lipoprotein cholesterol.

The CEA model examined the following two treatment alternatives for patients with hypertension and diabetes: a single-pill regimen with amlodipine/atorvastatin (referred to as single-pill) and a double-pill regimen with amlodipine and atorvastatin (referred to as double-pill). For each treatment alternative, the patients were classified into an adherent or non-adherent group depending on adherence. Ultimately, a CEA was performed, which incorporated the adherence rates observed for each treatment (Figure 1).

The analysis model of this study evaluated the cost-effectiveness of the use of amlodipine and atorvastatin in patients with diabetes who were on blood pressure-lowering medication to reach the target LDL-C level.

Effectiveness was defined as the proportion (%) of patients who attained the target LDL-C level with treatment. The target LDL-C level was <100 mg/dL, which is the level for high-risk patients in the National Cholesterol Education Program Adult Treatment Panel III (NCEP ATP III) guidelines [4]. The bio-equivalence of the two treatment alternatives has been demonstrated [10]. As such, any disparity between the effectiveness of the two is assumed to stem from patients’ adherence to the medications. Generally, medication adherence is described as proportion of days covered (PDC) or medication possession ratio (MPR), with a standard of 80% [14]. Therefore, adherence was defined as PDC ≥80%, and non-adherence as PDC <80%. The average cost-effectiveness ratios (ACERs) of annual cholesterol treatments for hypertensive patients with diabetes were analyzed. The treatment regimens were assumed to be once daily for a year, without variations in the amount or concentration. For each treatment, the ACER was calculated as the cost associated with 1% attainment of the treatment target.

### Data source

Table 1 displays the literature selected for this study. To determine the medication adherence rate, a systematic online literature search was conducted with “([amlodipine AND atorvastatin AND diabetes] AND adherence)” and “([amlodipine AND atorvastatin AND diabetes] AND compliance)” as key words. The search resulted in 19 and 39 relevant publications, respectively. Initially, the titles and abstracts were reviewed to exclude literature that did not meet the study purpose such as non-randomized clinical trials as well as patient groups and outcomes that did not meet the criteria. However, accurate effectiveness values for medication adherence rates in patients with diabetes were not available; therefore, we decided to estimate the effectiveness values based on research data of the medication adherence rates in the general patient population and subgroups of patients with diabetes. This required a re-examination of the search results, and two publications were selected: one pertaining to the adherence rates and effectiveness values in the general patient population [11] and the other presented odds ratios (ORs) of adherence rates in patients with diabetes [15].

Subsequently, another systematic online literature search was conducted with “diabetes AND LDL-C AND atorvastatin AND adherence” as key words to retrieve data pertaining to the target goal attainment based on adherence. The key words used in the previous literature search were used. Ultimately, one publication was selected [16] that analyzed the target LDL-C attainment rates based on the adherence rates in patients with diabetes and high cholesterol. The data for this study were extracted from the literature.

The medication costs were obtained from data published by the Health Insurance Review and Assessment Services. The weighted average medication costs of September 2014 were used for the price of the single-pill (5 mg amlodipine/10 mg atorvastatin). The 2013 annual weighted average medication costs by compound were used for the price of the double-pill (5 mg amlodipine and 10 mg atorvastatin).

### Medication adherence

Medication adherence rates in the general patient population [11] as well as the ORs for patients with diabetes [15] were used to estimate medication adherence rates for the two treatments in this study.

Adherence rates of 67.7 and 49.9% were used for the single-pill and double-pill in general patients, respectively [11]. For the medication adherence OR, a sensitivity analysis was performed using an unadjusted OR that reflected various factors, including diabetes, that potentially affect adherence rates (age, smoking status, and CVD history). In other words, a sensitivity analysis was performed that did not hold the factors constant, other than diabetes. Calculated adherence rates used for this analysis were 67.0 and 49.4% for the single-pill and double-pill in diabetes patients, respectively. An adjusted OR of 1.06 was used, for which the factors other than diabetes were also held constant (age, smoking status, and history of CVD) in this study.

### Target goal attainment rates

Because the bio-equivalence of the two treatments has been demonstrated, an equal proportion of patients with diabetes who attained the target LDL-C level were assumed for both of the treatments. According to the retrieved literature [16], the attainment rates were 56 to 78% and 16 to 42% for the adherent and non-adherent groups, respectively. For our analysis, the median values (adherent group, 67%; non-adherent group, 29%) were used. An additional sensitivity analysis was performed that incorporated the minimum and maximum attainment rates suggested in the literature.

### Costs

Medication costs reflecting adherence rates were assumed to be distributed near the top 80% of the adherence rate. In other words, for a patient in the adherent group, the adherence rate would be PDC≥80%, indicating that the patient adhered to the medication regimen for 292 to 365 days of the year. Therefore, it was assumed that the patient would adhere to the regimen an average 350 days per year, which is at the top 80% of the range. For a patient in the non-adherent group, the adherence rate would be <80%, indicating that the patient adhered to the regimen for 0 to 291 days of the year. Therefore, it was assumed that the patient would adhere to the regimen for an average of 232 days per year, which is at the top 80% of the range [17].

## RESULTS

The results of the economic analysis are expressed using CER, which integrates costs and clinical effectiveness. CER can be divided into ACER and the incremental cost-effectiveness ratio (ICER). ACER compares the ACER of one alternative with another, and the smaller the ACER, the more cost effective the alternative. ICER is an index used to identify the potential costs associated with improving the outcome by one unit; it is obtained by dividing the differences in outcomes by the differences in costs.

In this study, ACER was used. An base case analysis of the medication adherence and target goal attainment rates resulted in a 56.3% effectiveness value for the single-pill and 49.1% for the double-pill. The sensitivity analysis incorporating the minimum and maximum target goal attainment rates resulted in 44.7% for the single-pill and 37.2% for the double-pill with the minimum value and 67.8 and 61.0%, respectively, with the maximum value. The sensitivity analysis for the adherence rates effectiveness values (using an unadjusted OR based on the same target attainment rates) resulted in 54.5 and 47.8% for the single-pill and double-pill, respectively.

The assumption of medication costs around the top 80% range resulted in estimated medication costs of 256,550 Korean won (KRW) for the single-pill and 353,850 KRW for the double-pill. The base case analysis, which took into account the medication adherence rates, resulted in medication costs of 228,612 KRW for the single-pill and 294,082 KRW for the double-pill. A sensitivity analysis resulted in medication costs of 228,027 KRW for the single-pill and 293,486 KRW for the double-pill (Table 2).

### Cost-effectiveness

The results of an base case analysis indicated that a 1% improvement in the attainment rates incurred an additional 4,123 KRW for the single-pill and 6,062 KRW for the double-pill. This translates into an approximate 32% savings with the single-pill for the same level of outcome, indicating that the single-pill is a more efficient alternative than the double-pill (Table 3).

**Figure 2.**
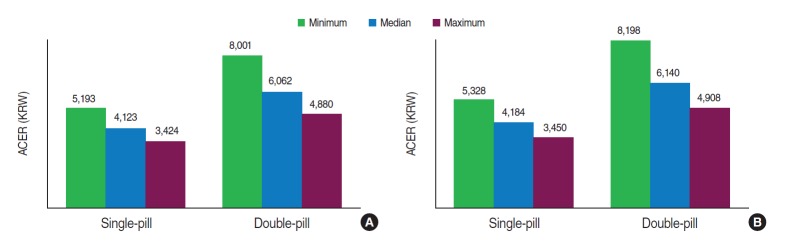
ACER based on adjusted odds ratios (A) and unadjusted odds ratios (B) in sensitivity analysis. ACER, average cost-effectiveness ratio; KRW, Korean won.

The sensitivity analysis regarding changes in target goal attainment rates revealed that, in both treatments, ACER decreased as the attainment rates increased, from 67 to 78%, with the same adherence rate used in the base case analysis: single-pill, from 4,123 to 3,424 KRW; double-pill, from 6,062 to 4,880 KRW) (Figure 2A). Regarding the single-pill and double-pill, the ACER of the single-pill was lower than that of the double-pill (3,424 vs. 4,880 KRW), indicating that the single-pill regimen is more efficient.

According to the sensitivity analysis using the unadjusted OR to calculate the medication adherence rates, the ACERs were higher than those in the base case analysis (single-pill, from 4,123 to 4,184 KRW; double-pill, from 6,062 to 6,140 KRW). Nevertheless, a similar pattern is preserved here, in which the single-pill shows lower ACERs than the double-pill (minimum, 5,328<8,198 KRW; median, 4,184<6,140 KRW; maximum, 3,450<4,908 KRW) (Figure 2B), indicating that the single-pill is the more efficient alternative.

In other words, all of the ACER findings indicate that the single-pill is more efficient in meeting the target goal than the double-pill.

## DISCUSSION

Improved standards of living and increasing interest in a healthy lifestyle are increasing the cost of healthcare. Incorporating medication costs into the existing effect, efficacy, and safety standards is critically important to ensure effective healthcare spending. Because a single combination pill costs less than separate pills for the same medications in the domestic marketplace, it could be assumed that the total medication cost of a single-pill would be lower than that of a double-pill. However, the transfer of potential savings into economic gains needs to consider medication adherence rates.

Medication adherence rates are expressed as PDC or MPR; both are calculated using the number of medications prescribed during the prescription period. However, MPR is expressed as a ratio of the prescription period and number of medications, whereas PDC is expressed as a percentage of the number of medications during the prescription period. PDC is thought to be more consistent than MPR, which is a relative figure [14,18]. Therefore, most research uses PDC to express medication adherence rates. An 80% cut-off point defines high and low adherence rates.

Assuming adherence to the medication by all patients, similar effectiveness and lower medication cost with a single-pill can be predicted easily without the need of a CEA. In reality, medication adherence rates vary depending on the patient characteristics and specific diseases. Therefore, a CEA that considers these differences are needed, which is a major limitation of this study.

This study aimed to determine, through a CEA model that considered adherence rates, a potential improvement in medication adherence rates and economic benefits of single-pill and double-pill prescribed to control the cholesterol levels of patients with diabetes and controlled hypertension.

The results indicated that, with each 1% improvement in the attainment rates, 4,123 KRW was required for the single-pill and 6,062 KRW for the double-pill, indicating that the single-pill is 32% less costly than the double-pill for the same outcome level. Also, for the sensitivity analysis of adherence and target goal attainment according to adherence, ACER decreases as the target goal attainment increase.

In 2013, cerebrovascular diseases, CVDs, and diabetes accounted for the second, third, and fifth causes of deaths in Korea, respectively [19]. Diabetes, in particular, is known to increase the mortality rates of CVDs by 2.5 to 4 times [20]. In patients with diabetes, hypertension is closely associated with CVDs, and approximately 80% of patients with diabetes have concomitant hypertension [21]. Hypertension accelerates the progression and increases the incidence of CVDs. According to the current diagnostic criteria, approximately 50 to 80% of patients with diabetes also have high cholesterol. Because high cholesterol is a risk factor for CVDs, proactive treatment is particularly important.

Most international examination guidelines recommend the evaluation of the overall cholesterol profile (total cholesterol, high density lipoprotein cholesterol, and LDL-C) [22-24]. Recently published Korean guidelines for hypertension management indicate that patients with diabetes are at a higher risk for CVDs and recommend that blood pressure should be maintained at <140/85 mmHg [25]. Other research also indicates that individuals with risk factors, such as diabetes and hypertension, should maintain LDL-C levels below the threshold to effectively prevent CVDs [26,27].

The NCEP ATP III, the US diagnostic guidelines for hyperlipidemia, use ranges of risk levels for the major CVD risk factors to base treatment recommendations for each risk level. According to these guidelines, diabetes considered a risk factor for coronary heart disease, and the LDL-C level should be maintained at 100 mg/dL.

According to a recently published Korean study of diabetes, 62.6% of patients with diabetes have LDL-C levels <100 mg/dL [28]. This low rate of cholesterol control underscores the importance of evaluating the effects of a single combination pill, as it may contribute to improved medication adherence rates. Korean studies that have compared the cost-effectiveness of a single-pill with that of a double-pill based on adherence rates are rare. A 2014 study compared the economic analysis, through improved medication compliance, of a single-pill combination therapy with a double-pill administration for the primary prevention of cardiovascular disorders in Korean patients with hypertension, hyperlipidemia, and at least 3 risk factors for CVDs [17]. The per-individual cost of CVD prevention was used to determine the cost-effectiveness. The results indicated that the single-pill prevented 0.4 to 4.5 CVDs per 1,000 individuals by improving the adherence rates. The final results also showed that the single-pill can result in 32% cost savings. This research is sorely needed because this CEA study, which evaluated the economic effects of the single-pill in patients with diabetes, controlled hypertension, and hyperlipidemia, is thought to help guide the selection of medications.

Patient with diabetes and controlled hypertension are at a greater risk for cerebrovascular diseases such as stroke, congestive heart failure, and peripheral vascular diseases. Therefore, proactive treatment and prevention are needed to control and maintain optimal blood pressure. Diabetes accompanied by dyslipidemia and hypertension increases the risk of CVDs even further; therefore, blood pressure-lowering and cholesterol-lowering agents should be administered to these patients. The single-pill, which integrated the two agents, might help to improve medication adherence rates.

Adherence rates generally decrease with an increasing number of medications. Therefore, a decrease in the number of medications is expected to improve adherence rates. The proportion of patients who successfully adhere to medication regimens doubles when they were on single-pill [29]. Lower adherence rates affect treatment outcomes, as evidenced by the superior treatment outcomes observed in the adherent group compared with the non-adherent group. The CEA also confirmed that the single-pill is a more efficient alternative than the double-pill.

This study has certain limitations, requiring cautious interpretation of the results. First, the probability of adherence and target LDL-C attainment rate were extracted from two different sources, which may have affected the accuracy of the estimations. Nevertheless, uncertainties were minimized by selecting data after a thorough review of applicable data through a systematic research. Second, all of the effect data were from research performed abroad with foreign patients. Therefore, future research on medication adherence and attainment rates that reflect the treatment situation in Korea is needed. Third, only the medication costs were incorporated into the CEA; therefore, the ability to interpret a total cost required to improve the attainment rates by 1% is limited. Fourth, because patients with diabetes and controlled hypertension who required dyslipidemia management were included, the attainment rates for target blood pressure and LDL-C levels might not be accurate. The number of patients with diabetes who have successful blood pressure management (40%) is lower than patients without diabetes (68%), according to the 2011 Korea National Health and Nutrition Examination Survey [30]. Future studies are needed to determine the target blood pressure and cholesterol attainment rates based on adherence rates. Fifth, because the outcome was defined as target LDL-C level attainment rates, rather than prevention of CVD; it is difficult to identify the degree to which the additional cholesterol management (in patients with diabetes and controlled hypertension) contributes to CVD prevention. Nevertheless, the Heart Protection Study reported that lower LDL-C levels have a positive effect on primary prevention of CVD in patients with diabetes [31]. Additionally, the Coronary Artery Diabetes Study reported that the daily administration of atorvastatin to reduce LDL-C levels in patients with diabetes reduced CVD incidence, including strokes [26]. Therefore, the LDL-C level is associated with CVDs. Future studies that examine the degree to which reduced LDL-C levels can lower the risk of CVDs, as well as the economic analysis, are needed.

Despite these limitations, the CEA of the treatment alternatives in clinical settings in this study demonstrated that a single- pill is more efficient than treatment with double-pill for patients with diabetes and controlled hypertension who also require dyslipidemia management. The results of this study could help to select the optimal medication regimen for these patients.

## Figures and Tables

**Table 1.** Source of data in the model from the systematic review

Author	Patients	Treatment	Adherence definition	Endpoint	Extracted data
Probability of adherence					
Patel et al. [11]	Adults taking a CCB or statin (but not both) who then initiated treatment with SPAA or added CCB to statin (or vice versa)	Co-administration vs. single-pill administration	PDC ≥80%	% of patients with PDC ≥ 80 %	ATR/AML: 67.7% ATR+AML: 49.9%
Chapman et al. [15]	Patients with co-morbid hypertension and dyslipidemia at high risk for cardiovascular disease	Co-administration vs. single-pill administration	PDC ≥80%	% of adherent patient	Diabetes adherence OR (vs. noncoronary artery disease) Adjusted OR (95% CI): 1.06 (0.96. 1.17) Unadjusted OR (95% CI): 0.99 (0.90 1.08)
LDL-C level according to adherence level					
Parris et al. [16]	Patients with diabetes and dyslipidemia	Statin	PDC ≥80%	LDL-C goal (<100 mg/ dL) attainment according to adherence level	PDC≥80% (MPR, %): 56-78 PDC < 80% (MPR. %): 16-42

CCB, calcium channel blocker; SPAA, single-pill amlodipine/atorvastatin; PDC, proportion of days covered; ATR, atorvastatin; AML, amlodipine; OR, odds ratio; CI, confidence interval; LDL-C, low density lipoprotein cholesterol; MPR, medication possession ratio.

**Table 2.** Drug cost for each medication regimen

Alternative	Compliance	Cost		Treatment cost (KRW)	Average cost (KRW)
		Unit price (KRW)	Treatment period (d)		
Single-pill (ATR/AML)	Adherent^1^	733	350	256,550	228,612
	Non-adherent	733	232	170,056	
Double-pill (ATR+AML)	Adherent^1^	1,011	350	353,850	294,082
	Non-adherent	1,011	232	234,552	

KRW, Korean won; ATR, atorvastatin; AML, amlodipine.

1 Adherent≥80% proportion of days covered: 2013 weighted average price used for unit price.

**Table 3.** Average cost-effectiveness ratios

	Compliance rate (%)	Target goal attainment (LDL-C level <100 mg/dL, %)	Probability (%)	Total cost (KRW)	ACER (KRW)
Single-pill (ATR/AML)	Adherence (71.8)	Attainment (67.0)	56.3	232.126	4.123
		Not attainment (33.0)			
	Non-adherence (28.2)	Attainment (29.0)			
		Not attainment (71.0)			
Double-pill (ATR+AML)	Adherence (52.9)	Attainment (67.0)	49.1	297.653	6.062
		Not attainment (33.0)			
	Non-adherence (47.1)	Attainment (29.0)			
		Not attainment (71.0)			

Modified from Patel BV, et al. Vasc Health Risk Manag 2008:4:673-681 [11].

LDL-C, low density lipoprotein cholesterol; KRW, Korean won; ATR, atorvastatin; AML, amlodipine.
