# Supplementary material for: Cost-effectiveness analysis of low density lipoprotein cholesterol-lowering therapy in hypertensive patients with type 2 diabetes in Korea: single-pill regimen (amlodipine/atorvastatin) versus double-pill regimen (amlodipine+atorvastatin)
Source: Epidemiol Health. 2015 Feb 22;37:e2015010. doi: 10.4178/epih/e2015010 (PMC4835702; doi:10.4178/epih/e2015010)
Supplement: Supplementary file 1 [file epih-37-e2015010-supplementary.pdf]

Cost-effectiveness Analysis of LDL cholesterol-Lowering Therapy in Hypertensive Patients with Type-2 Diabetes in Korea: Single-Pill Regimen (Amlodipine/Atorvastatin) versus Double-Pill Regimen (Amlodipine + Atorvastatin)

고혈압을 동반한 당뇨환자들의 LDL-콜레스테롤 관리의 비용-효과분석: 단일복합제제투여(Amlodipine/Atorvastatin)와 단일제제의 병용투여(Amlodipine + Atorvastatin)와의 비교

ABSTRACT

Objective: Single-pill combination therapy (Amlodipine/Atorvastatin) may be more effective compared with a Double-pill approach (Amlodipine+Atorvastatin) in hypertensive patients requiring concomitant statin therapy. We investigated the cost-effectiveness of Single-pill compared with Double-pill for prevention of cardiovascular disease (CVD) through additional controlling LDL-C (Low Density Lipoprotein Cholesterol) level with consideration to adherence in diabetes and hypertension patients using cost-effectiveness analysis model.

METHODS: We determined effectiveness as percentage (%) attainment of target LDL-C levels (< 100 mg/dL) which depends on differences in patients' adherence to each alternative. 'Adherence' was defined as compliance to medication over 80% on proportional day covered (PDC) and 'non-adherence' for the remaining. The proportion of the adherent patient and target goal attainment differences in adherence level were searched through systematic review. The annual costs of medicine were included according to the adherence levels of each alternative. Average cost-effectiveness ratio (ACER) was calculated as the cost per % attainment of target LDL-C level.

RESULTS: In this study, defined Average cost-effectiveness ratio (ACER) is the cost per 1% achievement of target goal (LDL-C < 100 mg/dL), as maintaining optimal LDL-C levels is crucial for the prevention of cardiovascular disease in patients with hypertension accompanied by diabetes. ACERs in Single-pill approach were lower than those in Double-pill approach (4,123 KRW vs. 6,062 KRW per 1 % achievement of target goal). Single-pill approach reduced medication cost by around 32 % than Double-pill approach.

Conclusion: Single-pill approach is cost effective compared with Double-pill approach in hypertensive patients with diabetes.

Key word: Atorvastatin, Amlodipine, Adherence, LDL-C level, Cost-effectiveness,

I. 서론

2011년에 발표된 문헌에 따르면 최근 한국에서 심혈관계 질환 유병률과 함께 이로 인한 사망률도 급격하게 증가하고 있으며 고혈압과 당뇨, 이상지질혈증, 비만, 등은 심혈관 질환의 주요 위험인자인 것으로 보고하고 있다[1]. 따라서 이들 위험인자들이 서로 동반될 경우 심혈관 질환의 위험이 더욱 증가하기 때문에 적극적인 관리가 매우 중요하다[2]. 특히 당뇨환자들의 경우에는 고혈압과 이상지질혈증이 동반되면 심혈관질환 발생 위험이 높아지기 때문에 혈압 조절과 동시에 엄격한 혈중 지질농도의 조절(LDL-C level < 100 mg/dL)을 통해 심혈관질환을 예방하도록 최근의 치료지침에서 권고하고 있다[3,4].

일반적으로 혈압과 혈중지질농도 목표치에 도달하는 환자 비율은 치료받은 환자의 절반 이하로 혈압과 혈중지질농도 조절률은 저조한 것으로 보고되었으며[5] 그 원인으로는 환자들의 낮은 복약 순응도를 생각할 수 있다[6]. 복약 순응도는 질병을 관리하고 치료하는데 있어서 약리학적으로 치료의 유용성에 영향을 주는 매우 중요한 요인으로[7] 약제의 개수 및 복잡한 복용방법과 관련이 있는 것으로 보고되고 있다. 따라서 환자의 복약 순응도를 높일 수 있는 방법으로 환자들의 복용 부담을 감소시킬 수 있는 단일 복합제 형태의 약제 사용을 들 수 있으며 실제 연구에서도 단일제제 병용투여에 비해 단일 복합 약제 투여가 환자의 복약 순응도를 증가시키고, 그 결과로 치료 성과에도 긍정적인 영향을 미친다는 결과가 보고된 바가 있다[8,9].

항고혈압제인 Amlodipine besylate(이하 Amlodipine)과 지질저하제인 Atorvastatin calcium(이하 Atorvastatin)의 단일복합제제의 치료효과는 단일제제의 병용투여 치료 효과와 동등(bio-equivalent)하며[10] 복약 순응도 측면에서 유의한 개선을 나타내어[11] 환자들의 혈압과 혈중지질농도의 치료목

표 도달률을 증가시킬 것으로 기대하고 있다.

국내 단일복합제제의 가격이 단일제제 병용투여의 약제 가격보다 더 저렴하여 치료를 위한 총 약제비용은 단일복합제제의 약제비가 더 낮을 것으로 예상하지만 복약순응도를 고려하게 되면 복약순응도가 낮은 환자는 약을 복용하지 않는 만큼의 약제비도 감소하고, 낮은 복약순응도는 치료목표 도달률에도 영향을 미치게 되므로 환자들의 복약순응도 개선이 실제 경제적 이득으로 이어지는지에 대한 평가가 필요하다. 따라서 본 연구에서는 고혈압을 동반한 당뇨환자에게 혈중지질의 추가적인 관리가 필요한 경우 단일복합제제(Amlodipine/Atorvastatin) 투여와 단일제제 병용(Amlodipine + Atorvastatin) 투여의 두 대안에서 복약 순응도를 고려한 비용-효과성을 비교 분석하였다.

비용-효과분석은 경제성평가의 분석법 중 하나이며 경제성평가는 보건의료 자원배분에 있어서 객관적으로 제시할 수 있는 합리적인 근거를 도출할 수 있는 여러 방법들 중 하나이다. 경제성평가 연구는 비용과 의약품의 효능/효과, 삶의 질(QoL: Quality of Life)을 고려하여 의약품의 가치를 평가하는 것으로 비교대안에 비하여 개선된 임상적 유용성 대비 추가적으로 소요되는 비용을 비교하여 가장 효율적인 대안을 찾아내는데 목적이 있다고 할 수 있다.

II. 방법

경제성평가 분석에서 사용하는 비용의 유형은 크게 의료비용, 비의료비용 등의 직접비용과 생산성 손실 비용에 의한 간접비용으로 나눌 수 있다[12]. 의료비용은 의사방문비용, 입원비, 약제비 등 의료기관 서비스를 이용하는데 소요된 비용과 의료기기 구입과 같은 지출 내용도 모두 포함하며, 비 의료비용은 의료이용에 수반되는 비용으로 간병비, 교통비, 환자 가족 보육비 등도

포함하는 비용이다. 마지막으로 생산성 손실비용은 질환 또는 질환으로 인한 조기사망으로 인해 사회생활을 하지 못함으로 발생하는 경제적 손실비용을 의미한다. 이들 비용 중에서 대부분의 경제성평가 연구에서는 직접적으로 소요되는 의료비용을 가장 많이 사용한다. 경제성 분석 에서 비용 측정 방법은 어느 평가 방법이나 동일하다고 할 수 있지만 성과(Outcome)를 어느 범주까지 보느냐에 따라서 비용-최소화 분석(CMA: Cost-Minimization Analysis), 비용-효과 분석(CEA: Cost-Effectiveness Analysis), 비용-효용 분석(CUA: Cost-Utility Analysis), 비용-편익 분석(CBA: Cost-Benefit Analysis)으로 나눌 수 있다[13].

비용-최소화 분석(CMA)은 효과가 동일한 수준이라고 인정될 때, 비용만을 비교하는 방법으로 비용이 가장 저렴한 대안이 가장 효율적인 대안이라고 할 수 있다. 비용-효과 분석(CEA)이나 비용-효용 분석(CUA)은 효과의 크기가 다를 때 각 대안간의 효과의 차이와 비용의 차이를 함께 고려하여 경제성을 평가하는 방법이다. 특히 비용-효용 분석(CUA)은 질보정생존연수(QALY: Quality Adjusted Life Years), 건강수명(HYE: Healthy Years Equivalents)과 같이 삶의 양적인 측면과 질적인 측면을 함께 반영한 지표를 사용하여 평가한다. 비용-편익 분석(CBA)은 건강수준의 개선, 생존기간의 연장과 같은 편익을 건강 그 자체를 나타내는 지표가 아닌 화폐단위로 계량화하여 평가하고 비교하는 방법이다[13].

본 연구에서는 각 대안별 효과의 차이와 비용의 차이를 함께 고려하여 비용-효과 분석을 수행하였고 다만, 비용 추정시 약제비용을 제외한 다른 비용들은 두 치료대안에서 모두 동일한 것으로 가정하여 최종적으로 약제비만을 고려하였으며 두 대안의 효과는 동일하며 효과의 차이는 복약순응도에 의해서만 차이가 나는 것이라고 가정하였다.

이처럼 경제성평가 분석은 효과, 비용 등 여러 정보들을 종합, 합성하여 비용 대비 효과를

확인하는 방법으로 주로 결정분석 모형(Decision Analytic Model)을 이용하여 모형을 구축하게 된다. 결정분석 모형(Decision Analytic Model)에는 결정수형모형(Decision Tree Model)과 마콥모형(Markov Model)이 일반적으로 사용되며 그 중에서도 본 연구는 결정수형 모형(Decision Tree Model)을 사용하여 분석하였다. 이는 각 대안에 따른 건강상태가 일어날 가능성과 이에 따른 비용 정보를 함께 결합하여 각 대안의 기대비용과 기대효과를 계산하는 방법이다.

이처럼 경제성평가는 여러 가정과 많은 연구 결과들의 결합이므로 불확실성은 반드시 존재한다. 이러한 불확실성을 확인하고 최소화하기 위하여 비용 또는 효과 추정에 사용된 변수에 대하여 민감도분석(Sensitivity analysis)을 시행하게 되며 본 연구에서는 복약 순응도와 복약 순응도에 따른 치료목표 도달률 변화에 대하여 각각 민감도 분석을 수행하였다.

비용-효과 모형

본 모형은 비용-효과 분석 모형으로 고혈압을 동반한 당뇨 환자들을 대상으로 치료대안은 Amlodipine/Atorvastatin의 단일복합제제 투여(이하 Single-pill)와 Amlodipine + Atorvastatin의 단일제제의 병용투여(이하 Double-pill)이다. 두 치료대안에서 약물 복약 순응도에 따라 순응군(Adherent Group)과 비순응군(Non-adherent Group)으로 각각 분류하였고, 이렇게 분류한 그룹에 복약 순응도에 따른 치료목표 도달률를 각각 반영하였으며 최종적으로 Single-pill 과 Double-pill 두 대안의 복약 순응도를 고려한 비용-효과를 분석하였다.(Figure 1)

앞서 언급하였듯이 당뇨환자들의 경우에는 고혈압과 이상지질혈증이 동반하여 발생하면 심혈관질환 발생 위험이 매우 높아지기 때문에 혈압 조절과 동시에 엄격한 혈중 지질농도의 조절(LDL-C level < 100 mg/dL)을 권고하고 있다. 따라서 본 모형에서는 고혈압을 동반한 당뇨

환자들은 고혈압 조절을 위하여 이미 항고혈압 치료제를 복용을 하고 있으며 추가로 이상지질혈증에 대한 관리가 필요한 환자의 LDL-C의 치료목표도달을 위한 Amlodipine과 Atorvastatin의 비용-효과성을 평가하였다.

본 연구에서의 효과의 정의는 약물 투약 후 LDL-C가 치료목표수치에 도달하는 환자 분율(%)로 하였다. 이때 치료목표수치는 The National Cholesterol Education Program Adult Treatment Panel III (이하 NCEP ATP III) 지침에 의거하여 고위험군의 치료목표 수치인 LDL-C level < 100 mg/dL로 하였다[14]. 두 치료 대안은 생물학적 동등성(bio-equivalence)이 입증되었으므로[15] 각 대안간의 효과의 차이는 복약 순응도에만 영향을 받는 것으로 하였으며, 일반적으로 복약순응의 기준은 Proportional day covered(PDC, 또는 MPR; medication possession ratio) 80 % 이상으로 볼 수 있으므로[16] 본 분석에서도 복약 순응군은 PDC > 80 %으로, 비순응군은 PDC ≤ 80 %으로 정의하였다. 이 때 본 연구는 고혈압을 동반한 당뇨환자들에게 혈중 지질농도 조절을 위한 1년 동안의 약물 치료의 평균 비용-효과비(ACER)를 분석하였으며 각 치료대안은 1일 1회 투여하여 투여기간은 1년으로 투여용량의 조절 없이 유지된다고 가정하였다.

이들의 분석을 통하여, 최종적으로 각 치료대안의 평균 비용-효과비(ACER: Average Cost-Effectiveness Ratio)를 산출하여 비교하였으며 평균 비용-효과비(ACER)는 1 %의 치료목표 도달을 위하여 소요되는 비용으로 정의하였다.

자료원

본 연구의 효과값은 복약순응도와 복약순응도에 따른 치료목표 도달률을 사용하였으며 최종적으로 선정된 문헌들은 Table 1과 같이 정리하였다. 각 치료대안의 복약순응 정도를 확인하기

위하여 “((Amlodipine AND Atorvastatin AND diabetes) AND adherence)”, “((Amlodipine AND Atorvastatin AND diabetes) AND compliance)”를 검색어로 하여 체계적 문헌고찰을 실시하였다. 그 결과 각각 19개, 39개의 문헌이 검색되었고 제목과 논문 요약을 일차적으로 검토하여 무작위배정 임상시험이 아니거나 해당 환자군이 아니거나, 해당 outcome이 아닌 문헌을 제외하였다. 하지만 최종적으로 정확한 당뇨환자에 대한 복약순응도 관련 효과값이 존재하지 않아 각 치료대안에 대하여 일반 환자군을 대상으로 하여 복약순응도 정도를 분석한 문헌과 당뇨환자들의 Subgroup 분석결과를 포함한 문헌을 사용하여 당뇨환자에 대한 복약순응도의 효과값을 추정하기로 하였다. 따라서 앞서 시행한 문헌검색 결과를 재검토하였고 최종적으로 일반 환자군을 대상으로 한 복약순응도 관련 효과값을 포함한 문헌(Bimal V Patel, 2008)과 일반환자 대비 당뇨환자의 복약순응도 오즈비(Odds Ratio, 교차비)값을 포함한 문헌(Richard H. Chapman, 2005)을 선정하였다.

다음으로 복약 순응도에 따른 치료목표 도달률의 효과값은 “Diabetes AND LDL cholesterol AND Atorvastatin AND Adherence” 를 검색어로 하여 체계적 문헌고찰을 수행하였다. 검색된 문헌에 대하여 앞서 수행한 문헌고찰에서의 제외기준과 동일한 기준으로 문헌을 제외하였으며 최종적으로 고지혈증을 동반한 당뇨환자들의 LDL-C 치료목표 도달률을 복약 순응도에 따라 분석한 문헌(Elizabeth S. Parris, 2005)을 선정하였고 해당 문헌에서 효과값을 추출하였다.

본 분석에서의 약제비용은 건강보험심사평가원에서 발표한 자료를 사용하였으며 Amlodipine 5mg/Atorvastatin 10 mg의 단일복합제제(Single-pill)의 가격은 2014년 9월 기준 동일성분 가중평균 약가 기준, 단일제제(Amlodipine 5mg, Atorvastatin 10 mg) 병용투여(Double-pill) 분석을 위한 약제비는 2013년 연간 성분별 가중평균가를 적용하였다.

복약순응도

본 분석에서의 복약순응도는 일반환자의 복약순응도[17]와 당뇨환자의 오즈비(Odds Ratio, 교차비)값[18]을 사용하여 당뇨환자들에 대한 두 치료대안의 복약순응도 효과값을 추정하였다. 우선 일반환자의 Single-pill 투약군에서 복약 순응군의 비율은 67.7 %, Double-pill 투약군에서의 복약 순응군은 49.9 %을 사용하였고[17], 복약 순응도에 대한 오즈비는 복약순응도에 영향을 줄 수 있는 여러 가지 요인들(연령, 흡연, 심혈관 질환 발병 여부, 등) 중 당뇨를 포함하여 여러 요인들을 보정하지 않은 오즈비값 (Unadjusted Odds Ratio) 즉, 당뇨 외에 복약 순응도에 영향을 줄 수 있는 다른 요인들을 보정하지 않은 값을 사용한 민감도 분석도 진행하였으며 이때 계산된 당뇨환자들의 복약 순응도는 각각 67.0 %, 49.4 %이다. 본 분석에서는 앞서 언급한 연령, 흡연, 심혈관 질환 발병 여부, 등의 여러 요인 중에서 당뇨를 제외한 다른 요인들을 보정한 값(Adjusted OR)인 1.06을 사용하였다[18].

치료목표 도달률

두 치료대안의 생물학적 동등성이 입증되었으므로 당뇨환자의 순응도에 따른 치료목표 도달률, 즉 당뇨환자에서 LDL-C level이 치료목표수치에 도달하는 환자 분율은 두 대안에서 동일하게 적용하였다. 체계적 문헌고찰을 통하여 최종 선정된 문헌[19]에서 치료목표 도달률은 복약 순응군에서는 56 % ~ 78 %, 비순응군에서는 16 % ~ 42 %로 범위로 나타났으며 본 분석에서는 해당 범위의 중간값(Median, 순응군: 67 % vs. 비순응군: 29 %)을 기본분석으로 진행하였다. 또한 범위로 제시된 치료목표 도달률의 최저값과 최고값을 적용하여 추가 민감도 분석을 수행하였다.

비용

환자들의 복약 순응도를 반영한 약제비는 복약순응 수준의 범위에서 평균 상위 80 % 수준에 분포 하는 것으로 가정하여 적용하였다. 즉, 본 연구에서 정의한 기준에 따라 복약순응 환자의 경우 복약순응도가 PDC 80 % 이상이므로 365일 중 292~365일은 약제를 복용한 것이고, 범위 내 상위 80 % 수준인 평균 약 350일을 복용한 것으로 가정하였다. 비순응 환자는 PDC 80 % 이하의 범위인 0 ~ 79 %의 범위에 속하므로 0 ~ 291일의 범위 내에서 약제를 복용한 것이고, 범위 내 상위 80 %인 평균 약 232일을 복용한 것으로 가정하였다[20].

III. 결과

경제성평가에서 결과 제시는 앞서 언급한 것처럼 비용과 효과를 결합한 형태인 비용-효과비(CER: Cost Effectiveness Ratio)로 나타낸다. 이때 비용-효과비(CER)에는 평균 비용-효과비(ACER: Average Cost-Effectiveness Ratio)와 점증적 비용-효과비(ICER: Incremental Cost-Effectiveness Ratio)가 있다. 평균 비용-효과비(ACER)는 두 대안이 있을 때 각 대안의 평균 비용-효과비(ACER)를 각각 구하여 비교하는 방법으로 그 값이 작은 대안일수록 비용-효과적이라고 할 수 있다. 점증적 비용-효과비(ICER)는 두 대안의 효과 차이를 비용의 차이로 나눈 값으로 효과 1단위 만큼 개선하는데 추가비용이 어느 정도 소요가 되는지를 나타내는 지표이다.

본 연구는 두 치료대안에 대하여 각 대안의 평균 비용-효과비(ACER)를 구하여 비교하는 방법으로 분석하였다. 당뇨환자의 복약 순응도와 복약 순응도에 따른 치료목표 도달률로 계산한 기본분석의 최종 효과값은 Single-pill에서 56.3 %, Double-pill에서 49.1 %로 나타났으며, 순응도에 따른 치료목표 도달률을 범위의 최저값과 최고값을 사용하여 계산한 민감도 분석에서는 최저값

사용시 Single-pill에서 44.7 %, Double-pill에서 37.2 %로 나타났고 최고값 사용시에는 각각 67.8 %, 61.0 %로 나타났다. 추가적으로, 동일한 치료목표 도달률을 기준으로 보정되지 않은 오즈비값(Unadjusted Odds Ratio)을 사용하여 복약순응도 효과값의 민감도 분석 결과 Single-pill에서 54.5 %, Double-pill에서 47.8 %로 나타났다.

앞서 언급하였듯이 약제비용은 복약순응 수준의 범위에서 평균 상위 80 % 수준에 분포 하는 것으로 가정하였고 그 결과 1년간의 예상약제비는 단일제제는 353,850 원, 복합제제는 256,550원이라고 할 수 있다. 최종적으로 복약 순응도를 반영하였을 때 두 대안의 약제비용은 기본분석에서 Single-pill은 228,612 원, Double-pill은 294,082 원으로 나타났으며, 민감도 분석에서의 약제비용은 Single-pill과 Double-pill에서 각각 228,027 원, 293,486 원으로 나타났다.

비용-효과비

본 연구에서는 효과단위당 비용 즉, 평균 비용-효과비(ACER)를 LDL-C의 목표수치(< 100 mg/mL)에 도달하는 환자 1 % 증가당 소요되는 비용으로 정의하였다. 즉, 평균 비용-효과비(ACER)값이 작을수록 치료목표 도달에 효율적인 대안임을 의미한다. 기본 분석 결과, Single-pill의 경우에는 치료목표 도달률 1 % 증가시 4,123 원, Double-pill의 경우에는 6,062 원으로, 동일한 효과를 가져오는데 Single-pill 복용이 Double-pill 복용과 비교하여 약 32 % 정도의 비용이 적게 드는 것으로 Single-pill 복용이 효율적인 대안임을 확인할 수 있다. (Table 3)

치료목표 도달률 변화에 대한 추가 민감도 분석 결과, 두 대안 모두에서 복약순응도가 기본분석과 동일한 복약순응도인 경우 치료목표 도달률이 증가할수록(67 % → 78 %) 평균 비용-효과비(ACER) 값은 작아지는 것을 확인 할 수 있다 (Single-pill: 4,123 원 → 3,424 원, Double-pill:

6,062 원 → 4,880 원) (Figure 2(A)). 또한 Single-pill 과 Double-pill 복용군의 분석결과를 비교해보면 Single-pill에서의 평균 비용-효과비(ACER)가 Double-pill의 평균 비용-효과비(ACER)보다 더 낮아 (3,424 원 vs 4,880 원) Single-pill 투약군이 치료목표 도달에 효율적인 대안임을 확인 할 수 있다.

두 번째로 당뇨환자들의 복약순응도 값을 산출하기 위하여 보정하지 않은 OR(Unadjusted OR) 값을 사용했을 때 민감도 분석결과, 기본분석 결과와 비교하여 각각의 평균 비용-효과비(ACER)는 높아졌지만(Single-pill: 4,123 원 → 4,184 원, Double-pill: 6,062 원 → 6,140 원) 동일한 치료목표 도달률을 기준으로 Single-pill 투약군이 Double-pill 투약군과 비교하여 평균 비용-효과비(ACER)가 더 낮은 경향성은 동일하게 나타나므로 (5,328 원 < 8,198 원, 4,184 원 < 6,140 원, 3,450 원 < 4,908 원) Single-pill 투약군이 더 효율적인 대안임을 확인 할 수 있다(Figure 2(B)).

즉, 환자의 치료목표 도달률이 증가할수록 각 치료 대안별 평균 비용-효과비(ACER) 값은 낮아지며 두 치료대안의 결과값을 비교한 결과 Single-pill 투약군이 치료목표도달에 더 효율적임을 확인하였다. 또한 복약순응도가 증가할수록 이를 반영한 효과값과 약제비는 모두 증가하지만 두 치료대안의 결과값을 비교한다면 평균 비용-효과비(ACER) 값은 기본분석과 앞서 언급한 민감도 분석결과는 동일하게 Single-pill 복용이 효율적인 대안임을 확인할 수 있다.

IV. 고찰

최근 생활수준의 향상과 건강한 삶에 대한 욕구가 증대되어 이로 인하여 의료비 지출이 가파르게 증가하는 추세이다. 따라서 보다 효과적인 의료비 지출을 위하여 기존의 의약품 평가의 주요 기준이 효능/효과 및 안전성에 관한 기준과 더불어 비용을 함께 고려한 의약품 평가가 매우 중요하다.

일반적으로 국내 단일복합제제의 가격이 단일제제 병용투여의 약제 가격보다 더 저렴하여 총 약제 소요비용은 단일복합제제 투약군에서 더 낮을 것으로 예상하지만 복약순응도를 고려하게 되면 약제비와 복약순응도에도 영향을 미치게 되어 환자들의 복약순응도 개선이 실질적인 경제적 이득으로 이어지는지에 대한 평가가 필요하다.

복약 순응도는 Proportional Day Covered (PDC) 또는 Medication Possession Ratio (MPR) 등으로 나타낸다. 두 방법 모두 “처방기간 동안 처방된 약물의 개수”로 계산한 값이지만 MPR은 처방기간과 처방약물의 비(Ratio)로 나타내며, PDC는 처방기관과 처방약물의 비를 백분율로 환산한 값(Percentage)으로 상대적인 값의 MPR에 비하여 PDC의 값의 일관성이 더 크다고 할 수 있다[21, 22]. 따라서 대부분의 연구에서 복약 순응도를 나타낼 때 PDC를 이용하며 80%값을 기준으로 하여 그 이상인 경우에는 복약 순응도가 높다고 평가하며 그 미만인 경우에는 복약순응도가 낮다고 평가한다.

본 연구에서 실질적인 복약 순응도를 고려하지 않고 모든 환자들이 처방 받은 대로 약물을 투여하는 것으로 가정한다면 효과는 동일하고, 총 약제비용은 단일복합제제의 약제비가 더 낮으므로 비용-효과분석을 수행하지 않더라도 투약비용이 저렴한 단일복합제제가 단일제제 병용투여 방법보다 더 효과적임을 예상할 수 있다. 하지만 실제 환자들의 복약 순응도는 환자의 특성 및 질환에 따라 달라질 수 있으므로 이를 반영하여 비용-효과분석을 수행하는 것이 타당하며 이에 대하여 고려하지 못한 점은 본 연구에서 주요 한계점이라고 할 수 있다.

따라서 본 연구는 고혈압을 동반한 당뇨환자들을 대상으로 혈압조절이 되고 있다는 가정하에 혈중지질의 추가적인 관리가 필요한 경우 Single-pill 투여(Amlodipine/Atorvastatin)와 Double-pill (Amlodipine+Atorvastatin)의 복약 순응도에 따른 비용-효과를 분석함으로써 앞서 언급한 복약순응도

개선과 경제적 이득에 대하여 평가하고자 하였다. 비용측면으로는 치료 대안 별로 복약 순응도를 고려한 1년 동안의 약제비를 포함하였으며, 효과에 대해서는 각 치료 대안별 복약순응도와 복약순응도에 따른 LDL-C 수치의 치료목표 도달률을 적용하였다.

분석 결과, Single-pill의 경우에는 치료목표 도달률 1 % 증가시 4,123 원, Double-pill의 경우에는 6,062 원으로, 동일한 효과를 가져오는데 Single-pill 투약군이 Double-pill 투약군에 비해 약 32 % 정도의 비용이 적게 드는 것으로 나타났다. 따라서 당뇨를 동반한 고혈압환자를 대상으로 Single-pill 투여 방법이 치료 목표 도달률 1 % 증가를 위하여 소요되는 비용이 Double-pill 투여방법보다 적어 비용-효과적 대안임을 확인하였다. 또한 복약 순응도와 복약 순응도에 따른 치료목표 도달률에 대하여 민감도 분석을 수행한 결과, 치료목표 도달률이 증가할수록 평균 비용-효과비(ACER) 값은 작아지며(Single-pill: 4,123 원 → 3,424 원, Double-pill: 6,062 원 → 4,880 원) Single-pill에서의 평균 비용-효과비(ACER)가 Double-pill의 평균 비용-효과비(ACER)보다 더 낮아 (3,424 원 vs 4,880 원) Single-pill 투약군이 치료목표 도달에 효율적인 대안임을 확인 할 수 있다. 그 다음으로 Unadjusted OR 값을 사용하여 복약 순응도를 계산한 민감도 분석결과, Single-pill 투약군이 Double-pill 투약군과 비교하여 평균 비용-효과비(ACER)가 더 낮은 경향성을 보이므로 앞에서의 분석결과와 동일하게 Single-pill 투약군이 더 효율적인 대안임을 확인 할 수 있다.

2013년 한국인의 사망 원인 중 뇌혈관질환과 심장질환, 당뇨로 인한 사망이 각각 사망원인 2, 3 위와 5 위를 차지하고 있으며[23] 특히 당뇨는 다른 심혈관계질환으로 인한 사망률을 2.5 ~ 4 배 증가시키는 것으로 알려져 있다[24]. 또한 당뇨환자에서 고혈압은 심혈관계 질환과 밀접하게 관련이 있으며 당뇨환자의 80 % 정도는 고혈압을 동반하고[25] 고혈압은 심혈관계 질환의 진행속도를 빠르게 하여 질환의 발병률도 증가시킨다. 고지혈증의 경우에는 당뇨환자들의 약 50 –

80 %의 환자가 현재의 진단 기준상 질환을 앓고 있는 것으로 보이고 있으며 고지혈증 또한 심혈관계 질환을 야기시키는 위험요인 중의 하나로 적극적인 치료가 반드시 필요하다. 이와 관련하여 대부분의 제 외국 진료지침에서 전체 지질평가(총 콜레스테롤, HDL 콜레스테롤, LDL 콜레스테롤)를 권고하고 있고[26-28] 최근 발표된 국내 고혈압 진료지침에서도 당뇨병을 위험인자로 분류하여 당뇨를 가진 환자들을 심혈관 고위험군으로 규정하여 고혈압의 경계치 미만(< 140 / 85 mmHg)으로 혈압을 조절하도록 권고하고 있다[29]. 또한 다른 여러 연구에서도 당뇨가 있거나, 고혈압과 동시에 다른 위험인자를 가지고 있는 고위험군에서 혈압과 LDL-C 수치를 정해진 역치 이하로 낮추는 것이 심혈관계 질환 예방에 효과적일 수 있음이 보고되었다[30, 31].

고지혈증 진료지침인 미국의 NCEP ATP III에 따르면 주요 심혈관계 위험인자에 따라 범주를 나누고 각 위험률의 범주에 따라 고지혈증의 치료방법을 권고하며 이 지침에 따르면 당뇨병을 CHD risk equivalents로 분류하여 LDL-C 수치를 100 mg/dL 이하로 낮출 것을 권고하고 있다.

최근 한국인 당뇨병 환자를 대상으로 발표된 연구에 따르면, 당뇨병 환자에서 LDL-C < 100 mg/dL으로 조절되는 비율은 62.6 % 정도이며[32], 이러한 낮은 조절률을 고려할 때, 복약 순응도를 향상시킬 수 있는 단일복합제의 투약의 효과를 평가하는 것은 중요하다고 할 수 있다. 하지만 복약순응도에 따른 단일제제의 병용투여와 단일복합제 투여의 비용-효과 분석의 국내 연구는 매우 드문 것이 현실이다. 2014년에 한국인을 대상으로 한 문헌에서 고지혈증이 동반된 고혈압 환자 및 위험인자 3개 이상의 심혈관질환 고위험군 환자군을 대상으로 심혈관질환의 일차예방을 위해 사용되는 단일복합제 투여와 단일제제의 병용투여의 순응도 개선에 따른 경제성을 비교하여 평가한 문헌[33]은 일반 환자에 대하여 단일복합제제와 단일제제 병용투여의 두 치료대안의 비용-

효과비를 산출하여 비교하였으며 이때 1명의 심혈관질환 예방을 위해 소요되는 비용으로 최종 비용-효과비를 확인하였다. 연구 결과, 단일복합제 투여시의 순응도 개선이 단일제제 병용투여와 비교하였을 때 1000명당 0.4~4.5명의 심혈관 질환 예방을 보이는 것으로 확인하였고, 최종적인 분석결과, 단일복합제 투여시 약 20% 비용절감을 가져오는 것으로 나타났다. 해당 문헌 외에 의료진이 실제 임상에서 약제 처방시 고려하는 치료대안들의 경제성을 평가한 문헌, 더욱이 한국인 당뇨환자들을 대상으로 한 비용-효과분석 문헌은 거의 존재하지 않아 이에 대하여 연구가 필요하였다. 그런 의미에서 본 연구에서 수행한 비용-효과 분석으로 단일복합제 투약이 고혈압을 동반한 당뇨환자들에게 추가적인 고지혈증 치료에 경제적으로 효과적인지를 평가하고 약제를 선택하는데 의미 있는 자료가 될 것이다.

다시 말해, 고혈압을 동반한 당뇨 환자들에게 혈압은 뇌졸중, 울혈성 심부전, 말초혈관질환과 같은 심뇌혈관질환의 주요 위험위인으로 뇌혈관질환 및 여러 합병증의 위험과 함께 이로 인한 사망률도 시키므로 혈압 조절을 위하여 보다 적극적인 치료가 필요하며 당뇨환자들의 경우에는 고혈압과 더불어 이상지질혈증이 동반되면 심혈관질환 발생 위험이 더욱 더 높아지므로 혈압 조절을 위한 혈압강하제와 함께 지질저하제의 투여가 필요하다. 이때 복약순응도를 개선한 혈압강하제와 지질저하제의 단일복합제의 투약이 중요한 역할을 할 수 있을 것이다.

앞서 언급하였듯이 본 연구는 고혈압을 동반한 당뇨환자들에게 추가적으로 이상지질혈증이 나타나 이에 대한 약물치료가 필요한 경우 이를 치료하기 위하여 단일제제 병용투여 방법과 환자의 복용편의성을 개선한 단일복합제제 투여방법에 대하여 비용-효과성을 평가하였다. 일반적으로 환자들은 복용하는 약제의 개수가 증가하면 복약순응도는 감소하는 경향을 보여 복용 약제의 개수를 감소시키는 것이 복약순응도 개선의 효과를 가져올 수 있을 것으로 기대하고 있다.

실제로도 단일복합제 투여시 복약순응 환자 비율이 단일제제 병용투여 환자비율보다 약 2 배정도 높은 것으로 보고되었다[34]. 이러한 복약순응도의 차이는 치료결과에도 영향을 미치게 되어 복약순응군에서의 치료효과가 비순응군의 효과와 비교하여 더 우수하며 비용-효과 분석 결과 단일복합제 투여군이 단일제제 병용투여군과 비교하여 더 효율적인 대안임을 본 분석 결과 확인할 수 있었다.

본 연구는 다음과 같은 제한점을 가지고 수행되었으므로 결과 해석시 주의가 필요하다. 첫째, 복약순응도에 대한 확률값과 복약순응도에 따른 LDL-C 치료목표 도달률의 효과값이 서로 다른 문헌에서 추출되어 정확한 결과를 추론하는데 한계가 있으며 이는 체계적 문헌고찰을 통하여 가용할 수 있는 모든 자료들을 검토하여 선정하였으므로 불확실성을 최소화 하였다. 둘째, 효과와 관련된 자료 모두가 외국인 환자를 대상으로 한 문헌으로부터 추출한 값이므로 국내 현실을 반영하는데 한계가 있다. 따라서 앞서 언급하였듯이 국내 치료 현실을 반영하여 치료 대안에 대한 복약순응도 및 치료목표 도달률에 대한 추가 연구가 필요할 것이다. 셋째, 비용 측면에서 약제비만을 분석에 포함하였으므로 최종적으로 치료목표 도달률 1 % 증가를 위하여 필요한 총 비용이라고 해석하기 어렵다. 넷째, 본 연구는 고혈압을 동반한 당뇨병 환자들에게 추가로 고지혈증 치료가 필요한 경우에 대하여 분석을 진행 한 것으로 혈압도달률과 콜레스테롤 도달률에 대한 정확한 결과를 추론하는데 한계가 있다. 당뇨병 환자에서의 혈압 조절도 비당뇨병 환자와 비교시 그 비율은 낮은 편으로 2011년 국민건강영양조사 연구에 따르면, 혈압약을 복용하는 환자 중에 혈압이 적정 수준으로 조절되는 비율은 비당뇨병 환자에서 68 %, 당뇨병 환자에서 40 %에 불과하다고 보고하였다[34]. 추후 각 치료대안에 대하여 복약순응도에 따른 혈압과 이상지질혈중 농도 모두의 치료목표 도달률이 어느 정도 인지에 대한 추가 연구가 필요할

것이다. 다섯째, 본 연구에서는 이상지질혈증의 추가 치료가 필요한 고혈압을 동반한 당뇨환자들의 치료 목표를 심혈관질환 예방이 아닌 LDL-C level의 치료목표 도달률로 정의하였으므로 구체적으로 고혈압을 동반한 당뇨환자들에게 고지혈증의 추가 치료가 심혈관질환 예방을 어느 정도 예방할 수 있는지 확인하기에 한계가 있다. 하지만 HPS 연구(Heart Protection Study)에서 LDL-C 감소가 당뇨병 환자의 심혈관 질환의 일차 예방에 효과가 있고[36] CARDS(Coronary Artery Diabetes Study) 연구에서도 당뇨병 환자들을 대상으로 LDL-C 농도 감소를 위하여 Atorvastatin을 매일 투여한 결과 뇌졸중으로 포함한 심혈관계 질환의 발생이 의미 있게 감소하였다고 보고하였으므로[37] LDL-C level은 심혈관계 질환 발생과 연관이 있다고 할 수 있다. 추후 해당 환자군과 각 치료대안에 대하여 LDL-C 감소가 구체적으로 심혈관질환 발생을 어느 정도 예방할 수 있으며 이에 대한 비용-효과는 어떠한지에 대하여 추가 연구가 필요할 것이다.

이와 같은 여러 제한이 있음에도 불구하고 본 연구는 한국인 당뇨환자들을 대상으로 하여 실제 임상에서 고려하는 치료대안들의 비용-효과분석을 수행한 것으로 고혈압을 동반한 당뇨환자들이 추가적으로 고지혈증 치료가 필요한 경우 단일복합제 투약이 더 효과적인 대안임을 확인한 문헌이다. 따라서 본 연구결과가 해당 환자들이 약제를 선택하는데 하나의 중요한 참고가 될 수 있을 것으로 기대된다.

참고문헌

1. Kim YJ, Kwak CY, Prevalence and Associated Risk Factors for Cardiovascular Disease: Finding from the 2005, 2007 Korea National Health and Nutrition Examination Survey, Korean J Health Promot. 2011;11(3):169-176
2. Asia Pacific Cohort Studies Collaboration. Joint Effects of Systolic Blood Pressure and Serum Cholesterol on Cardiovascular Disease in the Asia Pacific Region. Circulation 2005;112:3384-3390.
3. Grundy SM, Balady GJ, Criqui MH, Fletcher G, Greenland P, Hiratzka LF, et al., Primary prevention of coronary heart disease: guidance from Framingham: a statement for healthcare professionals from the AHA Task Force on Risk Reduction. Circulation 1998;97:1876-1887
4. Expert Panel on Detection, Evaluation, and Treatment of High Blood Cholesterol in Adults (Adult Treatment Panel III): Executive summary of the Third Report of the National Cholesterol Education Program (NCEP). JAMA 2001;285:2486-2497
5. Johnson ML, Pietz K, Battleman DS, Beyth RJ. Therapeutic Goal Attainment in Patients With Hypertension and Dyslipidemia. Medical Care 2006;44(1): 39-46.
6. Cowie MR. Simultaneous treatment of hypertension and dyslipidemia may help to reduce overall cardiovascular risk: focus on amlodipine/ atorvastatin Single-pill therapy. Int J Clin Pract 2005;59(7): 839-846.
7. Cramer JA. Partial medication compliance: the enigma in poor medical outcomes. American Journal of Managed Care 1995;1(2):167-174.
8. Dezii CM. Medication noncompliance: What is the problem? Managed Care 200;9 Suppl:7-12.
9. Simpson SH, Eurich DT, Majumdar SR, Padwal RS, Tsuyuki RT, Janice V, et al. A meta-analysis of the association between adherence to drug therapy and mortality. BMJ 2006;333(15):E1–E6.
10. Chung M, Calcagni A, Glue P, Bramson C. Bioavailability of Amlodipine besylate/Atorvastatin clacium combination tablet. J Clin Pharmacol 2006; 46(9):1030-1037.
11. Patel BV, Leslie RS, Thiebaud P, Nichol MB, Tang SS, Solomon H, et al., Adherence with Single-pill Amlodipine/Atorvastatin vs a two-pill regimen. Vascular Health and Risk Management 2008:4(3):673-681.
12. Kim CM, Health Economics and Outcomes Research, Korean J Fam Med 2009;30:577-587
13. Kim CM, Pharmacoeconomics, Kor J Clin Pharmacol Ther 2008;16(1):3-12

14. Expert Panel on Detection, Evaluation, and Treatment of High Blood Cholesterol in Adults (Adult Treatment Panel III): Executive summary of the Third Report of the National Cholesterol Education Program (NCEP). JAMA 2001;285:2486–2497
15. Chung M, Calcagni A, Glue P, Bramson C. Bioavailability of Amlodipine besylate/Atorvastatin clacium combination tablet. J Clin Pharmacol 2006;46(9):1030-1037.
16. Nau DP, Proportion of Days Covered(PDC) as a Preferred Method of Measuring Medication Adherence, Available from: http://www.pqaalliance.org/files/PDCvsMPRfinal.pdf
17. Patel BV, Lesile RS, Thiebaud P, Nicol MB, Tang SSK, Solomon H, et al., Adherence with single-pill amlodipine/atorvastatin vs a two-pill regimen, Vascular Health and Risk Management 2008;4(3):673-681
18. Chapman RH, Benner JS, Pertilla AA, Teirce JC, Collins SR, Battleman DS, et al., Predictors of Adherence With Antihypertensive and Lipid-Lowering Therapy, Arch Intern Med. 2005;165:1147-1152
19. Parris ES, Mohn LA, Lawrence DB, Long LB, Adherence to Statin Therapy and LDL Cholesterol Goal Attainment by patients with Diabetes and Dyslipidemia, Diabetes Care 2005;28:595-599
20. Kim YJ, Ko SK, Lee CW, Cost-Effectiveness of Single-Pill Combination Therapy of Amlodipine/Atorvastatin Compared with Concurrent Two-Pill Therapy in Patients with Hypertensive in Korea., J Health Tech Assess 2014;2(1):26-34
21. Nau DP, Proportion of Days Covered(PDC) as a Preferred Method of Measuring Medication Adherence, Available from: http://www.pqaalliance.org/files/PDCvsMPRfinal.pdf
22. Hess LM, Raebel MA, Conner DA, and Malone DC, Measurement of Adherence in Pharmacy administrative Databases: A Proposal for Standard Definitions and Preferred Measures, Ann Pharmacother 2006;40:1280-1288
23. Korea national statistical office. The number of major cause of deaths 2013. Available from: http://kostat.go.kr/portal/korea/kor_nw/2/1/index.board?bmode=read&aSeq=330181
24. Roper NA, Bilous RW, Kelly WF, et al,. Excess mortality in a population with diabetes and the impact of material deprivation: longitudinal, population based study. BMJ 2001;322:1389-1393
25. Cha BS, Treatment of Hypertension in Diabetes patients, Available from: www.Zonedoctor.co.kr (Korean, authors' translation)
26. Evidence Based Guidelines for Type 2 Diabetes: Macrovascular disease. Canberra: Diabetes Austraia & NHMRC, 2004. Available from: http://www.diabetesaustralia. com.au

27. Canadian Diabetes Association Clinical Practice Guideline Expert Committee: Canadian Diabetes Association 2003 Clinical Practice Guideline for the Prevention and Management of Diabetes in Canada. Canadian Journal of Diabetes 2003;27[Suppl.2]:S58-S65

28. Mclntosh A, Hutchinson A, Feder G, Durrington P, Elkeles R, Hitman GA, et al., Clinical guidelines and evidence review for Type 2 diabetes: Lipid Management. Sheffi eld:ScHARR, University of Sheffield, 2002 Available from: http://www.nice.org.uk/pdf/lipidsfull_guideline.pdf

29. 2013 Hypertension Treatment Guideline

30. Colhoun HM, Betteridge DJ, Durrington PN, Hitman GA, Neil HA, Livingstone SJ, et al., Primary prevention of cardiovascular disease with atorvastatin in type 2 diabetes in the Collaborative Atorvastatin Diabetes Study (CARDS): multicenter randomized placebo-controlled trial. Lancet 2004;364:685-696

31. Sever PS, Dahlof B, Poulter NR, Wedel H, Beevers G, Caulfield M, et al., Prevention of coronary and stroke events with atorvastatin in hypertensive patients who have average or lower-than-average cholesterol concentrations, in the Anglo-Scandinavian Cardiac Outcomes Trial-Lipid Lowering Arm (ASCOT-LLA): a multicenter randomized controlled trial. Lancet 2003;361:1149-1158

32. Roh E., Ko SH, Kwon HS, Kim NH, Kim JH, Kim CS, et al., Prevalence and Management of Dyslipidemia in Korea: Korea National Health and Nutrition Examination Survey during 1998 to 2010, Diabetes Metab. J 2013;37:433-449.

33. Kim YJ, Ko SK, Lee CW, Cost-Effectiveness of Single-Pill Combination Therapy of Amlodipine/Atorvastatin Compared with Concurrent Two-Pill Therapy in Patients with Hypertensive in Korea., J Health Tech Assess 2014;2(1):26-34

34. Champman RH, Yeaw J, Roberts CS, Association between adherence to calcium-channel blocker and statin medications and likelihood of cardiovascular events among US managed care enrollees. BMC Cardiovasc Disord 2010;10:29

35. Ko SH, Kwon HS, Kim DJ, Kim JH, Kim NH, Kim CS, et al., Higher Prevalence and Awareness, but Lower Control Rate of Hypertension in Patients with Diabetes than General Population: The Fifth Korean National Health and Nutrition Examination Survey in 2011, Diabetes Metab. J. 2014;38:51-57

36. Heart Protection Study Collaborative Group. MRC/BHF Heart Protection Study of Cholesterol-lowering with simvastatin in 5963 people with diabetes: a randomized placebo-controlled trial. Lancet 2003;361: 2005-2016

37. Colhoun HM, Betteridge DJ, Durrington PN, Hitman GA, Neil HA, Livingstone SJ, et al., Primary prevention of cardiovasdular disease with atorvastatin in Type 2 diabetes in the collaborative Atorvastatin Diabetes Study (CARDS): a multicenter randomized controlled trial. Lancet 2004;364:685-696.
